# An association between systolic blood pressure and stroke among patients with impaired consciousness in out-of-hospital emergency settings

**DOI:** 10.1186/1471-227X-13-24

**Published:** 2013-12-17

**Authors:** Taro Irisawa, Taku Iwami, Tetsuhisa Kitamura, Chika Nishiyama, Tomohiko Sakai, Kayo Tanigawa-Sugihara, Sumito Hayashida, Tatsuya Nishiuchi, Tadahiko Shiozaki, Osamu Tasaki, Takashi Kawamura, Atsushi Hiraide, Takeshi Shimazu

**Affiliations:** 1 Department of Traumatology and Acute Critical Medicine, Osaka University Graduate School of Medicine, 2-5 Yamada-oka, Suita, Osaka 565-0871, Japan; 2 Health Services, Kyoto University, Yoshida-Honmachi, Sakyo-ku, Kyoto 606-8501, Japan; 3 Department of Social and Environmental Medicine, Graduate School of Medicine, Division of Environmental Medicine and Population Sciences, Osaka University, 2-5 Yamada-oka, Suita, Osaka 565-0871, Japan; 4 Department of Trauma and Critical Care Medicine and Burn Centers, Social Insurance Chukyo Hospital, 1-1-10 Sanjyo Minamiku, Nagoya, Aichi 457-8510, Japan; 5 Osaka Municipal Fire Department, 1-12-54 Kujo minami, Nishi-ku, Osaka 550-8566, Japan; 6 Department of Critical Care and Emergency Medicine, Osaka City University Graduate School of Medicine, 1-5-17 Asahimachi, Abeno-ku, Osaka 545-8585, Japan; 7 Nagasaki University Hospital Emegency Medical Center, 1-7-1, Sakamoto, Nagasaki, Nagasaki 852-8501, Japan; 8 Department of acute Medicine, Kinki University Faculty of Medicine, 377-2 Ouno higashi Osaka-Sayama, Osaka 589-8511, Japan

**Keywords:** Systolic blood pressure, Prehospital, Impaired consciousness

## Abstract

**Background:**

Stroke is difficult to diagnose when consciousness is disturbed. However few reports have discussed the clinical predictors of stroke in out-of-hospital emergency settings. This study aims to evaluate the association between initial systolic blood pressure (SBP) value measured by emergency medical service (EMS) and diagnosis of stroke among impaired consciousness patients.

**Methods:**

We included all patients aged 18 years or older who were treated and transported by EMS, and had impaired consciousness (Japan Coma Scale ≧ 1) in Osaka City (2.7 million), Japan from January 1, 1998 through December 31, 2007. Data were prospectively collected by EMS personnel using a study-specific case report form. Multiple logistic regressions assessed the relationship between initial SBP and stroke and its subtypes adjusted for possible confounding factors.

**Results:**

During these 10 years, a total of 1,840,784 emergency patients who were treated and transported by EMS were documented during the study period in Osaka City. Out of 128,678 with impaired consciousness, 106,706 who had prehospital SBP measurements in the field were eligible for our analyses. The proportion of patients with severe impaired consciousness significantly increased from 14.5% in the <100 mmHg SBP group to 27.6% in the > =200 mmHg SBP group (*P* for trend <0.001). The occurrence of stroke significantly increased with increasing SBP (adjusted odd ratio [AOR] 1.34, 95% confidence interval [CI] 1.33 to 1.35), and the AOR of the SBP > =200 mmHg group versus the SBP 101-120 mmHg group was 5.26 (95% CI 4.93 to 5.60). The AOR of the SBP > =200 mmHg group versus the SBP 101-120 mmHg group was 9.76 in subarachnoid hemorrhage (SAH), 16.16 in intracranial hemorrhage (ICH), and 1.52 in ischemic stroke (IS), and the AOR of SAH and ICH was greater than that of IS.

**Conclusions:**

Elevated SBP among emergency patients with impaired consciousness in the field was associated with increased diagnosis of stroke.

## Background

Stroke is an important public health problem in the industrialized world [1] and there are 300,000 estimated strokes encounter in the prehospital settings annually Japan [2]. To improve neurologic outcomes after stroke, earlier identification and treatment is most important, but it takes longer time for EMS personnel to transport emergency stroke patients to the stroke centers if EMS personnel could not appropriately recognize these patients [3]. If EMS personnel can discriminate patients with stroke in prehospital settings, these patients can be transported fast to appropriate hospitals that offer advanced treatments such as thrombolytic therapy and interventional radiology.

Importantly, it is difficult to assess neurological findings such as paralysis of stroke in patients with impaired consciousness, and an alternative way to select these patients would, therefore, be needed. Although a lot of studies have showed the positive association between systolic blood pressure (SBP) and the risk of stroke occurrence [4], little is known about the relationship between SBP measured by EMS personnel and the risk of stroke occurrence among patients with impaired consciousness.

Osaka City is a largest urban community in western Japan with approximately 2.7 million population, and approximately 200,000 ambulance runs documented annually since January 1998. The purpose of this study was to evaluate the relationship between SBP measured by EMS in prehospital settings and stroke occurrence among emergency patients with impaired consciousness.

## Methods

### Study design, population, and setting

This is a retrospective, population-based observational study based on the ambulance records of Osaka Municipal Fire Department. The study period was from January 1, 1998 to December 31, 2007. This study was approved by the Ethics Committee of Kyoto University Graduate School of Medicine.

All adult patients aged > =18 years who suffered impaired consciousness, and were transported to medical institutions by EMS in Osaka City were enrolled in this study. Diagnoses of stroke and its subtypes such as subarachnoid hemorrhage (SAH), intracranial hemorrhage (ICH), and ischemic stroke (IS) were clinically determined by the physicians caring for the patients in collaboration with the EMS personnel.

### Japan Coma Scale

Table 1 shows Japan Coma Scale (JCS) for grading impaired consciousness [5]. The level of consciousness among emergency patients was recorded by EMS personnel according to JCS. The JCS is a simple way for evaluating neurological disturbance focused on patient’s awareness. EMS personnel have generally been using it in prehospital settings. The JCS was roughly divided into the three categories (e.g., mild disturbance, moderate disturbance, and severe disturbance).

**Table 1:** Japan Coma Scale for grading impaired consciousness

Mild disturbance:	
The patient is awake without any stimuli, and is	
1.	Almost completely conscious,
2.	Unable to recognize time, place and person,
3.	Unable to recall name and date of birth.
Moderate disturbance:	
The patient can be aroused	
10.	Easily by being spoken to,
	*(responsive with purposeful movements, phrases, or word)
20.	With loud voice or shaking the shoulders,
	*(almost steadily responsive with very simple words-yes or no, or movements)
30.	Only by repeated mechanical stimuli.
Then, the patient falls into the previous state by cessation of stimulation.	
Severe disturbance:	
The patient cannot be aroused by any forceful mechanical noise stimuli, and	
100.	Responds with movements to avoid the stimulus,
200.	Responds with slight movements including decerebrate and decorticate postures,
300.	Does not respond at all except for change in respiratory rhythm.

*Used in patients who cannot open their eyes for any reason.

This table was revised from Reference [5].

### Emergency medical service systems and hospitals in Osaka City

Osaka City, which is a largest urban community in western Japan, has an area of 222 km^2^, and its population was approximately 2.7 million in 2000 (population density, approximately 12,000 persons/km^2^). The municipal EMS system has been previously described [6]. Briefly, the EMS system is operated by the Osaka Municipal Fire Department and activated by dialing 119 on the telephone. There were 25 fire stations and a dispatch center in 2007 in Osaka City [7]. Life support is provided 24 hours every day. Usually, each ambulance has a crew of three emergency providers including at least one Emergency Life-Saving Technician (ELST), a highly-trained prehospital emergency care provider. Osaka City included 194 hospitals (34,209 beds) in 2007. Of them, 90 hospitals including 5 critical care centers can accept patients transported by ambulance [8].

### Data collection and quality control

Data were uniformly collected using the specific forms that included sex, age, location, vital signs such as first documented systolic and diastolic blood pressure measured manually with sphygmomanometer, heart rate, respiratory rate, and oxygen saturation. The diagnosis was determined by the physician responsible for the care of the patient before admission in the emergency department. The data form was filled out by the EMS personnel in cooperation with the physicians caring for the patient, transferred to the EMS Information Center of Osaka Municipal Fire Department, and then checked by the investigators. If the data sheet was incomplete, the investigators returned it to the relevant EMS personnel for data completion.

### Statistical analysis

The association between the occurrence risk of stroke and SBP (every 20 mmHg) was “*a priori*” analyzed considering its subtype (SAH, ICH, or IS). Patient characteristics with and without SBP measurements were evaluated with the use of the *t-*test for numeric variables and the chi-square test for categorical variables. Trends in categorical values and numerical values were tested with logistic regression models and linear tests for trend, respectively. Multiple logistic regression analysis was used to assess the occurrence risk of stroke and its subtype among emergency patients with impaired consciousness by 20 mmHg stratum; Adjusted Odds ratios (AORs) and their 95% confidence intervals (CIs) were calculated. Potential confounding factors were sex, age, and level of consciousness. In addition, the relationship between prehospital SBP and stroke occurrence by impaired consciousness level was evaluated. Statistical analyses were performed with SPSS statistical package version 17.0 J (SPSS, INC., Chicago, IL). *P* value of <0.05 was considered statistically significant.

## Results

**Figure 1:**
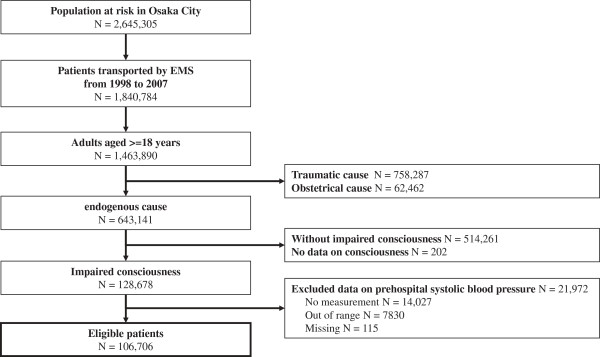
**Study flow of emergency patients from January 1, 1998 to December 31, 2007.** EMS: emergency medical service.

During these 10 years, a total of 1,840,784 emergency patients were documented during the study period in Osaka City (Figure 1). Among 1,463,890 adult patients, 643,141 had medical causes excluding obstetrical and trauma causes, 128,678 yielded an impaired consciousness, and 106,706 with prehospital SBP data were eligible for our analyses.

Table 2 shows the characteristics between 106,706 patients with SBP value and 21,972 patients without SBP value. In patients with SBP, mean men age was 63.1 years, and 54.2% were male. The proportion of mild impaired consciousness was 70.7%, moderate impaired consciousness 15.9%, and severe impaired consciousness 13.4%, respectively. Forty-nine percent of patients with impaired consciousness were assessed in a private residence. Mean initial SBP was 139.5 mmHg. Time interval from call to hospital arrival was 25.1 minutes. Although there were statistically significant differences because of the very large number, the characteristics between the groups were almost similar.

**Table 2:** Characteristics of eligible and non-eligible patients

	**Eligible**	**No eligible**	**P value**
	**(N = 106,706)**	**(N = 21,972)**	
Men, n (%)	57,879 (54.2)	13,341 (60.7)	<0.001
Age, year, mean (SD)	63.1 (20.7)	62.3 (20.4)	<0.001
> = 75 years, n (%)	37,793 (35.4)	7167 (32.6)	<0.001
Consciousness, n (%)			<0.001
Mild disturbance	75,437 (70.7)	12,573 (57.2)	
Moderate disturbance	16,979 (15.9)	2121 (9.7)	
Severe disturbance	14,290 (13.4)	7278 (33.1)	
Home, n (%)	52,936 (49.6)	10,164 (46.3)	<0.001
SBP, mmHg, mean (SD)	139.5 (36.1)	―	
DBP, mmHg, mean (SD)	77.8 (21.1)	―	
HR, counts per a minute, mean (SD)	89.0 (22.3)	70.7 (39.9)	<0.001
SpO_2_, %, median (IQR)	96 (94–98)	95 (88–98)	<0.001
Call to arrival at the scene, minute, mean (SD)	6.3 (2.5)	6.1 (2.5)	<0.001
Call to contact with patients, minute, mean (SD)	7.5 (2.7)	7.2 (2.8)	<0.001
Call to hospital arrival, minute, mean (SD)	25.1 (8.6)	22.3 (8.7)	<0.001

SBP denotes systolic blood pressure, DBP diastolic blood pressure, HR heart rate;

SD standard deviation, IQR inter-quartile range.

Table 3 shows the characteristics of eligible patients with impaired consciousness by prehospital SBP. As a whole, the proportion of patients with severe impaired consciousness significantly increased from 14.5% in the <100 mmHg SBP group to 27.6% in the > =200 mmHg SBP group (*P* for trend <0.001). Because there was an increase on the proportion of severe disturbance from 10.6% in the 101–120 mmHg group to 14.5% in the = <100 mmHg suggesting that low BP might be a factor in the altered mentation, the group with 101–120 mmHg SBP was defined as a reference group to show the relationship between prehospital SBP and stroke occurrence among patients with impaired consciousness.

**Table 3:** Characteristics of patients with impaired consciousness according to prehospital systolic blood pressure

	**SBP (mmHg)**						
	**= < 100**	**101-120**	**121-140**	**141-160**	**161-180**	**181-200**	**> = 201**
	**(N = 14,410)**	**(N = 22,352)**	**(N = 23,776)**	**(N = 19,465)**	**(N = 12,970)**	**(N = 7791)**	**(N = 5942)**
Age, year, mean (SD)	64.0 (21.0)	57.2 (22.4)	59.5 (22.3)	65.2 (19.1)	68.7 (15.9)	70.1 (14.1)	69.9 (13.2)
> = 75 years, n (%)	5485 (38.1)	6743 (30.2)	7639 (32.1)	7294 (37.5)	5243 (40.4)	3212 (41.2)	2177 (36.6)
Men, n (%)	7434 (51.6)	11,221 (50.2)	12,962 (54.5)	11,227 (57.7)	7357 (56.7)	4312 (55.3)	3366 (56.6)
Consciousness, n (%)							
Mild disturbance	9915 (68.8)	16,547 (74.0)	17,705 (74.5)	14,081 (72.3)	8948 (69.0)	4977 (63.9)	3264 (54.9)
Moderate disturbance	2408 (16.7)	3425 (15.3)	3608 (15.2)	3014 (15.5)	2092 (16.1)	1392 (17.9)	1040 (17.5)
Severe disturbance	2087 (14.5)	2380 (10.6)	2463 (10.4)	2370 (12.2)	1930 (14.9)	1422 (18.3)	1638 (27.6)

SBP denotes systolic blood pressure, SD standard deviation.

The proportions of patients with or without stroke according to the SBP were noted in Table 4. Among patients with impaired consciousness, 31.0% had the proportion of stroke (SAH 1.5%, ICH 6.3%, and IS 23.2%, respectively). This significantly increased from 17.1% to 63.7% (*P* for trend <0.001). The trends by the subtype of stroke were qualitatively similar.

**Table 4:** Proportion of stroke patients with impaired consciousness according to prehospital systolic blood pressure

		**SBP (mmHg)**						
	**All**	**= < 100**	**101-120**	**121-140**	**141-160**	**161-180**	**181-200**	**> = 201**
	**(N = 106,706)**	**(N =14,410)**	**(N =22,352)**	**(N =23,776)**	**(N =19,465)**	**(N =12,970)**	**(N =7791)**	**(N =5942)**
Stroke, n (%)	33,084 (31.0)	2467 (17.1)	4515 (20.2)	6050 (25.4)	6752 (34.7)	5506 (42.5)	4008 (51.4)	3786 (63.7)
SAH, n (%)	1631 (1.5)	81 (0.6)	124 (0.6)	243 (1.0)	285 (1.5)	270 (2.1)	286 (3.7)	342 (5.8)
ICH, n (%)	6699 (6.3)	228 (1.6)	434 (1.9)	709 (3.0)	1100 (5.7)	1260 (9.7)	1274 (16.4)	1694 (28.5)
IS, n (%)	24,754 (23.2)	2158 (15.0)	3957 (17.7)	5098 (21.4)	5367 (27.6)	3976 (30.7)	2448 (31.4)	1750 (29.5)
No stroke, n (%)	73,622 (69.0)	11,943 (82.9)	17,837 (79.8)	17,726 (74.6)	12,713 (65.3)	7464 (57.5)	3783 (48.6)	2156 (36.3)

SBP denotes systolic blood pressure, SAH subarachnoid hemorrhage, ICH intracranial hemorrhage, IS ischemic stroke.

**Figure 2:**
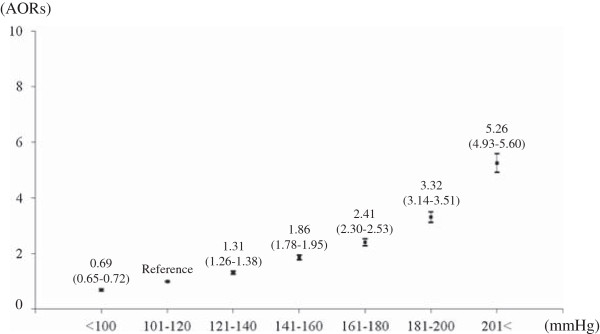
**Relationship between SBP measured by EMS personnel in prehospital settings and the risk of stroke occurrence among patients with impaired consciousness.** AORs: adjusted odds ratios.

**Figure 3:**
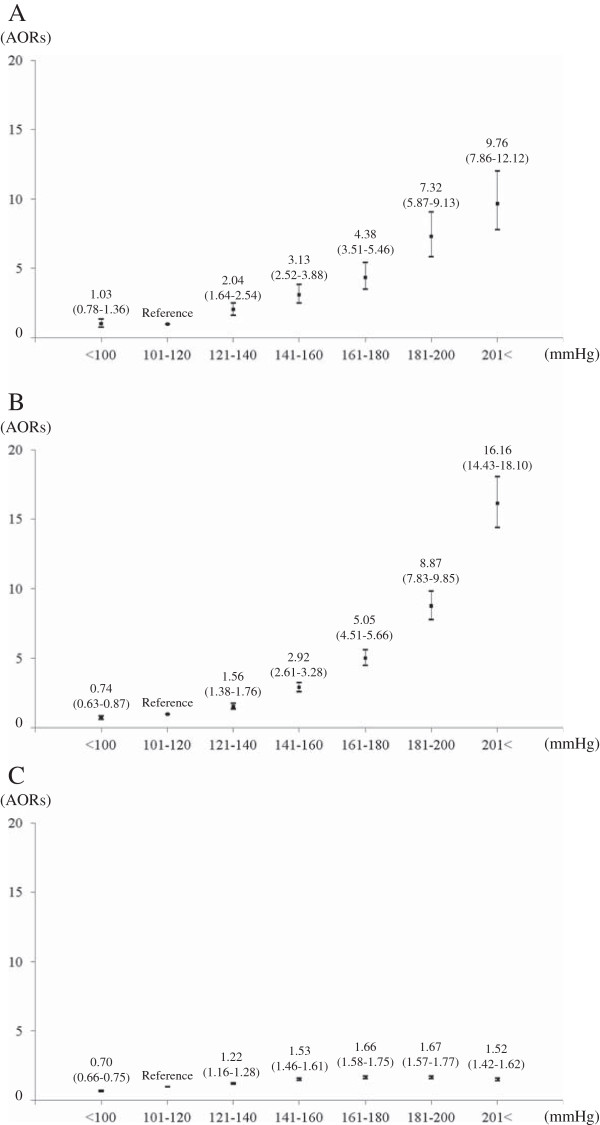
**Relationships between SBP measured by EMS personnel in prehospital settings and the risk of stroke occurrence by its stroke subtype among patients with impaired consciousness. (A)** SAH, **(B)** ICH, and **(C)** IS. AORs; adjusted odds ratios; SAH: subarachnoid hemorrhage; IS: ischemic stroke; ICH: intracranial hemorrhage.

Figure 2 shows the relationship between SBP measured by EMS in prehospital settings and stroke occurrence among patients with impaired consciousness. The occurrence of stroke significantly increased with increasing SBP (AOR 1.34, 95% CI 1.33 to 1.35), and the AOR of the SBP > =200 mmHg group versus the SBP 101-120 mmHg group was 5.26 (95% CI 4.93 to 5.60). In the subgroup analyses in the Figure 3, the AOR for 20 mmHg-increment of SBP was 1.48 (95% CI 1.43 to 1.52) in SAH, 1.69 (95% CI 1.66 to 1.72) in ICH, and 1.14 (95% CI 1.13 to 1.15) in IS, and the AOR of SAH and ICH was greater than that of IS. The AOR of the SBP > =200 mmHg group versus the SBP 101-120 mmHg group was 9.76 (95% CI 7.86 to 12.12) in SAH, 16.16 (95% CI 14.43 to 18.10) in ICH, and 1.52 (1.42 to 1.62) in IS, and the AOR of SAH and ICH was greater than that of IS.

Table 5 shows the relationship between prehospital SBP and stroke occurrence by impaired consciousness level. The AOR of the SBP > =200 mmHg group versus the SBP 101-120 mmHg group was 16.84 (95% CI 11.71 to 24.21) in mild disturbance and 11.55 (95% CI 6.70 to 19.90) in moderate disturbance among SAH patients, and 21.19 (95% CI 17.86 to 25.13) in mild disturbance, 13.58 (95% CI 10.71 to 17.22) in moderate disturbance, and 12.61 (95% CI 10.35 to 15.35) in severe disturbance among ICH patients.

**Table 5:** Relationship between prehospital SBP and stroke occurrence by impaired consciousness level

		**SBP (mmHg)**						
		**= < 100**	**101-120**	**121-140**	**141-160**	**161-180**	**181-200**	**> = 201**
	Mild disturbance	0.70 (0.65-0.75)	Reference	1.30 (1.23-1.38)	1.78 (1.68-1.88)	2.31 (2.17-2.45)	2.98 (2.78-3.20)	4.30 (3.96-4.66)
Stroke	Moderate disturbance	0.66 (0.58-0.75)	Reference	1.29 (1.16-1.44)	2.05 (1.84-2.29)	2.55 (2.26-2.87)	3.57 (3.12-4.08)	6.69 (5.72-7.82)
	Severe disturbance	0.70 (0.61-0.80)	Reference	1.39 (1.23-1.56)	2.03 (1.80-2.29)	2.75 (2.42-3.12)	4.80 (4.15-5.56)	8.21 (7.04-9.56)
	Mild disturbance	0.99 (0.60-1.63)	Reference	2.35 (1.66-3.34)	3.75 (2.64-5.32)	5.91 (4.13-8.45)	8.91 (6.14-12.94)	16.84 (11.71-24.21)
SAH	Moderate disturbance	1.01 (0.50-2.09)	Reference	2.34 (1.36-4.03)	3.59 (2.10-6.15)	5.08 (2.93-8.78)	9.92 (5.81-16.93)	11.55 (6.70-19.90)
	Severe disturbance	0.98 (0.66-1.46)	Reference	1.72 (1.23-2.39)	2.50 (1.81-3.45)	3.12 (2.25-4.32)	5.35 (3.89-7.37)	6.23 (4.58-8.48)
	Mild disturbance	0.74 (0.57-0.96)	Reference	1.76 (1.47-2.10)	3.17 (2.67-3.76)	5.82 (4.91-6.90)	10.90 (9.17-12.94)	21.19 (17.86-25.13)
ICH	Moderate disturbance	0.45 (0.31-0.67)	Reference	1.14 (0.88-1.48)	2.68 (2.11-3.39)	4.64 (3.67-5.87)	7.19 (5.66-9.14)	13.58 (10.71-17.22)
	Severe disturbance	0.88 (0.68-1.13)	Reference	1.62 (1.31-2.01)	2.80 (2.28-3.43)	4.35 (3.55-5.33)	7.34 (5.98-9.00)	12.61 (10.35-15.35)
	Mild disturbance	0.70 (0.65-0.75)	Reference	1.23 (1.16-1.30)	1.59 (1.47-1.65)	1.77 (1.67-1.89)	1.83 (1.70-1.97)	1.75 (1.60-1.90)
IS	Moderate disturbance	0.70 (0.61-0.81)	Reference	1.26 (1.12-1.41)	1.65 (1.47-1.86)	1.63 (1.44-1.85)	1.68 (1.46-1.93)	1.84 (1.58-2.15)
	Severe disturbance	0.67 (0.58-0.78)	Reference	1.15 (1.00-1.31)	1.27 (1.12-1.45)	1.26 (1.10-1.44)	1.16 (1.00-1.34)	0.91 (0.78-1.06)

SBP denotes systolic blood pressure, SAH subarachnoid hemorrhage, ICH, intracranial hemorrhage, IS ischemic stroke.

Odds ratio were adjusted for sex and age.

## Discussion

From this large registry of ambulance records, we demonstrated a significant positive relationship between prehospital SBP and the risk of stroke occurrence among emergency patients with impaired consciousness. Although little attention has been paid to SBP measured by EMS in prehospital settings in terms of diagnostic information for stroke, this large population-based registry enabled us to evaluate the relationship between prehospital SBP and stroke occurrence among these patients, and would add new insights on the importance of prehospital SBP measurement. Our results also suggest that prehospital SBP measurements in the patient with impaired conscious level might be a helpful guide as to where to transport a patient especially in communities that have both comprehensive stroke centers and primary ischemic stroke centers.

This study showed that the risk of stroke occurrence among emergency patients with impaired consciousness increased with increasing prehospital SBP. A previous study showed that initial SBP at emergency department arrival was of help for diagnosing intracranial lesion of patients with impaired consciousness [9]. However, diagnosis after hospital arrival is too late to transport the stroke patient to appropriate institution and start treatments against stroke in the effective time window [10]. Guidelines for the Early Management of Adults With Ischemic Stroke by American Heart Association recommend quicker transportation of suspected stroke patients to stroke care units to improve better neurological outcome [11]. Importantly, paralysis of stroke patients is frequently difficult to evaluate when their consciousness is disturbed. Therefore, this study showing the association between prehospital SBP measurements and stroke occurrence among patients with impaired consciousness would contribute to earlier detection of stroke and subsequent rapid transport to appropriate hospitals that can conduct specific treatments for them.

In analyses by stroke subtype, increased SBP was more strongly associated with occurrence of stroke among patients with hemorrhagic brain lesions such as SAH and ICH. The mechanism of hypertensive response among stroke patients is unclear [12] although patients with acute stroke and those with increased intracranial pressure often have hypertension. It was reported that 84% of patients with stroke had increased blood pressure in the acute phase [13]. The arterial pressure elevation in response to cerebral ischemia is known as the central nervous system ischemic response [14]. In ischemic stroke, hypertension maybe adaptive response to improve perfusion to the ischemic penumbra protecting the brain from further ischemia. On the other hand, hypertension in hemorrhagic brain lesion like SAH or ICH may cause further damage by worsening cerebral edema, increasing intracranial pressure, or causing hematoma expansion [15,16]. Our result showing difference by the subtype of stroke might be partially explained by such pathophysiological differences between hemorrhagic and ischemic lesions.

From our results, emergency patients with impaired consciousness and high SBP should be considered to be transported to the comprehensive stroke centers with capabilities of either neurosurgery or tissue plasminogen activator (t-PA) administration because these patients might have stroke but prehospital EMS personnel could not distinguish brain hemorrhagic lesions from ischemic ones. In addition, this study showed the strong relationship between high prehospital SBP and the occurrence of SAH and ICH, and those patients should be treated as quick as possible in order to prevent re-rupture of aneurysms and recurrent bleeding [17,18]. Especially, the strength of association between SBP and stroke subtype by impaired conscious level was very powerful with ICH and to some extent with SAH (mild and moderate disturbances), which would suggest that prehospital SBP can be an important triage guide for selecting patients. Further studies identifying an accurate cutoff point in this regard for SBP in conjunction with level of consciousness would make the EMS triage decision more precise and reduces the risk of overwhelming comprehensive stroke centers with patients that do not need the advanced capabilities. On the other hand, the relationship between SBP in prehospital settings and the occurrence of IS was relatively small. Therefore, to improve positive predictive value for IS patients who are most treatable, development of additional clinical indicators should be found out to make it possible to transport patients with IS to the primary ischemic stroke centers where only t-PA administration could be performed.

There were some limitations to this study. First, in Japan, EMS personnel evaluated level of consciousness among prehospital emergency patients by using JCS rather than the commonly-used Glasgow Coma Scale (GCS). JCS is not preferable to GCS as a consciousness evaluation system in the acute phase of SAH [19]. However, traditionally in Japanese prehospital setting, EMS has been evaluating stroke patients with impaired consciousness by JCS. Although our study cannot compare with GCS directly, these results should, nevertheless, provide useful information on the relationship between SBP and stroke occurrence among patients with impaired consciousness. Second, this study did not obtain data on advanced treatments and neurological outcomes among stroke patients after hospital arrival. Third, data on patient’s past history and medications that might affect the occurrence of stroke was lacking. Fourth, we did not obtain information on other diagnosis that could mimic stroke such as hypoglycemia, complicated migraine, prolonged seizures, and subdural hematomas. Finally, there might be unmeasured confounding factors and selection bias that might have influenced the relationship between prehospital SBP and stroke occurrence among emergency patients with impaired consciousness.

## Conclusions

Elevated SBP among emergency patients with impaired consciousness in the field was associated with the increased risk of stroke. Additional research is necessary to determine if field diagnosis of stroke with measuring BP improves procession of care or outcome.

## Abbreviations

SBP: Systemic blood pressure; EMS: Emergency medical service; AOR: Adjusted odds ratio; CI: Confidence interval; SAH: Subarachnoid hemorrhage; IS: Ischemic stroke; ICH: Intracranial hemorrhage; JCS: Japan come scale; ELST: Emergency life-saving technician; CIs: Confidence intervals; GCS: Glasgow Come Scale.

## Competing interests

The authors declare that they have no competing interests.

## Authors’ contributions

TI, TI, TK, CN, TS, KT, SH, TN, TS, OT, TK, AH, and TS participated in the idea formation, study design, data analyses, interpretation of results and writing of the report. All the authors read and approved the final manuscript.

## Pre-publication history

The pre-publication history for this paper can be accessed here:

http://www.biomedcentral.com/1471-227X/13/24/prepub
